# Live Birth Rate Comparison Between Single vs. Double Ovary Women With Assisted Reproduction: A Single Tertiary Center Study

**DOI:** 10.7759/cureus.14876

**Published:** 2021-05-06

**Authors:** Ghadeer L Aljahdali, Fatimah A Alkhaldi, Sarah F Almujarri, Haifa F Alsadhan, Amirah S Yaqoub, Jawaher A Alsahabi, Nazish Masud, Afaf A Felemban

**Affiliations:** 1 College of Medicine, King Saud bin Abdulaziz University for Health Sciences, Riyadh, SAU; 2 Research, King Abdullah International Medical Research Center, Riyadh, SAU; 3 College of Medicine, King Saud Bin Abdulaziz University for Health Sciences, Riyadh, SAU; 4 Department of Obstetrics and Gynecology, King Abdulaziz Medical City, Riyadh, SAU; 5 Research Unit, King Saud Bin Abdulaziz University for Health Sciences, Riyadh, SAU; 6 Department of In-Vitro Fertilization, King Abdulaziz Medical City, Riyadh, SAU

**Keywords:** in vitro fertilization iv, live birth rate, reproductive endocrinology, single ovary

## Abstract

Introduction

One of the major hardships faced by married couples is the inability to conceive a child. This issue is becoming more prevalent given the increasing rate of infertility worldwide. Assisted reproductive technology (ART) has brought hope to infertile couples. We aim to estimate the live birth rate (LBR) and pregnancy rate in women with one ovary compared with those with two ovaries.

Methods

A retrospective cohort study of women who underwent ART at King Abdulaziz Medical City (Jan 2000 - Dec 2018) was conducted. Five cycles of patient data were collected. The LBR (both conditional and cumulative) was compared between women with one and two ovaries.

Results

The final analysis included 403 women. Of these, 9% (n = 37) had one ovary. The majority (59%, n = 233) had primary infertility. A male-associated factor accounted for 52% (n = 208) of the infertility cases. The total number of live births was 164; and the overall LBR from five cycles was estimated as 9%, 16%, 18%, 18%, and 15%, respectively. In the double ovary group, the highest rate was in the fourth cycle [19% (12-26)], while in the single ovary group peaked in the third cycle [27% (9-46)]. Pregnancy was at its highest in the first cycle, accounting for 88 pregnancies.

Conclusion

The outcomes of ART varied between study groups. LBR was lower in single ovary women. The average of five cycles in the single and double ovary groups was 13% and 15%, respectively. Nevertheless, there was no significant difference in LBR between single or double ovary women.

## Introduction

Infertility is “a disease characterized by the failure to establish a clinical pregnancy after 12 months of regular, unprotected sexual intercourse or due to an impairment of a person's capacity to reproduce either as an individual or with his/her partner” [1]. Infertility is common worldwide with a prevalence of 8-12% [2]. According to the World Health Organization, there has been a significant worldwide decline in fertility rates over the past few decades. The World Bank’s database also shows a drastic decline in fertility rates from 5.05% in 1964 to 2.43% in 2017 [3]. Regional statistics in Saudi Arabia indicate a decline in fertility rates from 7.25% in 1994 to 2.37% in 2017 [3]. Infertile couples tend to seek help via assisted reproductive technologies (ART) such as in vitro fertilization (IVF), intra-cytoplasmic injection (ICSI), ovulation induction (OI), and intrauterine insemination (IUI). A Brazilian study conducted in 2019 estimated the prevalence of IVF and ICSI was 70.6% and 11.7%, respectively, compared to other technologies [4]. As infertility rates increasing globally, increased demand for ART has been observed. Providing patients with realistic expectations in terms of potential outcomes are mandatory before they go through the rigors of ART. The main goal of using ART is to conceive a live newborn and the probability of doing so is reflected upon the live birth rate (LBR).

Assisted reproductive technologies suitability and outcomes are dependent on many factors including age, type of infertility, sperm characteristics, hormone levels, history of gynecological pathologies and procedures, body mass index (BMI), and previous ART complications [5]. Moreover, conditions such as endometriosis, ovarian mass or cyst (benign or malignant), and complications (e.g., ectopic pregnancy) predispose to invasive procedures such as oophorectomy, salpingectomy, and hysterectomy which could affect IVF cycle outcomes and the length of treatment [6]. Locally, a study in Jeddah, identified the indications for laparoscopy as infertility (40%), ectopic pregnancy (15.8%), and endometriosis (11.1%) [7]. These will also affect ART outcomes such as pregnancy rate and LBRs, which are of particular concern when treating women with a single ovary. It remains unclear whether there are any ramifications in terms of ART outcomes. Among the scarce data comparing women with one and two ovaries, some studies have reported that the pregnancy rate is similar between the two groups, while others have found it to be less in single ovary women [8-9]. Similarly, others reported lower LBRs among women who underwent a unilateral oophorectomy (18.6%) compared with women who have two functional ovaries (25.4%) [10].

Assisted reproductive technologies outcomes include LBR, cumulative LBR (CLBR), and pregnancy rate (PR). Each rate has its importance and implications. Success rates for ART cycles have traditionally been reported as pregnancies per cycle according to maternal age. In most cases, success reporting in ART involves calculating outcome parameters such as pregnancy, delivery, (live) birth per started cycle, per (oocyte) aspiration, or per (embryo) transfer. LBR is an outcome that manifests the success of IVF and ICSI; furthermore, it is the primary outcome of the number of deliveries that result in live-born neonates [11]. The CDC reported that 284,385 ART cycles were performed in 2017, resulting in 68,908 live births in the United States [12].

In Saudi Arabia, there is a huge effort and demand for ART, yet there is a vast knowledge gap that must be bridged. We need a comprehensive study assessing ART success rates, techniques, and risk factors. Our study aimed to compare pregnancy, LBR, and CLBRs among women with single and double ovaries.

## Materials and methods

Study design, area, and settings

In this retrospective cohort study conducted at King Abdulaziz Medical City (KAMC), Riyadh, Saudi Arabia, we scrutinized the data from patients' health records (electronic and hard copy) from the In Vitro Fertilization (IVF) Unit and Health Information Management (HIM) system. This study was approved by the Institutional Review Board of King Abdullah International Medical Center (KAIMRC), Riyadh, Saudi Arabia.

Study participants, sampling technique, and sample size

We included Saudi women admitted between January 2000 and December 2018 to the IVF unit who underwent IVF or ICSI during the study period. Inclusion criteria included primary or secondary infertility, double or single ovary women, and normal hormonal levels such as prolactin or thyroid-stimulating hormone (TSH). Patients were excluded for documentation issues such as missing data. We used a stratified random sampling technique. We divided the subjects who met the inclusion criteria into two groups, double and single ovary women. In the literature, the LBRs in single and double ovary women were 18-25% and 32.3%, respectively [10]. Based on these previous studies, we calculated our sample size using a power of 80% and alpha 0.05; we assumed unequal values by keeping P1 > P2. Therefore, the estimated sample for double and single ovary groups were 300 and 132, respectively. However, we could not reach the estimated sample size for the single ovary group.

Data collection

Data were collected in collaboration with the In Vitro Fertilization and Health Information Management (HIM) departments, both of which are responsible for maintaining up-to-date records of all assisted fertility patients. Patient data from 2000 to 2015 were obtained from paper files at HIM department, while data from 2016 to 2018 were extracted from the hospital’s electronic database “BESTCare system”. Patients were followed during their ART trials, and the main variables included were wife and husband demographic profiles, duration of infertility, and semen analysis for men (volume, concentration, motility, morphology, viscosity, gelatinous particles, and spermatocyte). The grouping variables were single and double ovary groups and the presence of a male-associated factor as the cause of infertility. Aside from the underlying cause of infertility, pre-cycle details included type of ART, reproductive hormone levels, clinical investigations (hysteroscopy, ultrasound, laparoscopy, hysterosalpingogram), and treatments (Clomid, follicule-stimulating hormone [FSH], gonadotropin releasing hormone (GnRH), and human chorionic gonadotropin [hCG] supplements). Data for the first five cycles for all eligible women were extracted and analyzed.

Operational definitions

The pregnancy rate was defined as the number of pregnancies achieved ending with a viable birth with the exclusion of abortions. Live births reflect the number of deliveries that resulted in a live newborn. Three different live birth rates were analyzed: conditional, conservative, and optimal. Calculation details are provided in the supplementary file. The conditional live birth rate was calculated by dividing the number of live births per cycle by the number of women in that cycle. Conservative live birth rate (CLBR) is the cumulative live births over women's number in the first cycle [13-14].

Statistical analysis

After data entry was complete, the initial frequencies were run to check for any data entry deficiencies. Necessary corrections were made after accessing the original files when needed. Data were analyzed using the Statistical Package for Social Sciences (SPSS) version 22 and Microsoft Excel. Patient profiles are summarized in tables using percentages and frequencies for the categorical variables (e.g., ART, treatment, investigation, and presence of comorbidities). Numerical variables (e.g., age, number of cycle/oocytes/embryos, infertility duration) were presented as mean and standard deviation. The two groups were based on the number of ovaries. The primary outcomes (e.g., LBR, OLBR, CLBR) were presented in tables along with standard error. The LBR was calculated by dividing the total number of women who became pregnant by the eligible women who underwent ART in the corresponding cycle. The optimal live birth rate (OLBR) included women from the first cycle and other cycles. Kaplan-Meier curves were used to report the optimal estimate of the cumulative live birth rate, whereas the Greenwood formula was used to estimate the standard error. All of the estimates are presented in figures for all patients and stratification according to their respective groups [14-16].

## Results

Among women admitted to the IVF department, 846 were randomly chosen. Double and single ovary women accounted for 50% (n = 424) and 49.9% (n = 422), respectively. From which, 403 were included in this study, 9% (n = 37) single ovary while 91% (n = 366) double ovaries. Primary infertility accounted for 59% (n = 233). Infertility duration ranged from 24 to 168 months. Infertility due to male-associated factors accounted for 52% (n = 208). Hypothyroidism was the most prevalent comorbidity, 17% (69). A group of women (n = 91) had additional anovulatory cycles. On the other hand, the husband's mean age 35 ± 8 years. The presence of infertility problems accounted for 34% (n = 136). Comorbidities included spermatic disease 11% (n = 44), testicular disease 10% (n = 39). Semen analysis was also recorded in comparison between positive and negative male associated factors. Among husbands presenting with a male-associated factor, 26% (n = 54) received treatment, 27% (n = 57) testicular sperm aspiration (TESA). Tables 1-2 show wife and husband demographics respectively.

**Table 1:** Baseline Characteristics – Wife *From single ovary group. **Values reported based on the categorical variable “Yes”.

Wife Demographics	
Variables	N (%)
Infertility type	
Primary	233 (59%)
Secondary	164 (41%)
Infertility duration (months) Median (Q1-Q3)	48 (24-168)
Presence of male-associated factor	
Absent	195 (48%)
Present	208 (52%)
Presence of female-associated factor	
Motility (inhospitable)	87 (44%)
Oocyte	1 (1%)
Endometriosis	15 (8%)
Tubular	53 (27%)
Uterine	11 (6%)
Hormonal	5 (3%)
Unexplained	25 (13%)
Ovarian	3 (2%)
Ovary Number	
Single	37 (9%)
Double	366 (91%)
Surgery Type^*^	
Salpingoophrectomy	4 (31%)
Salpingectomy	3 (23%)
Oopherectomy	5 (39%)
Partial oophorectomy	1 (8%)
Wife Comorbidities^**^	
Hypothyroidism	69 (17%)
Hyperprolactinemia	31 (8%)
Dyslipidaemia	5 (1%)
Diabetes	16 (4%)
Hypertension	5 (1%)
Bronchial asthma	10 (3%)
Others	9 (4%)

**Table 2:** Baseline characteristics – husband *Values reported based on categorical variable “Yes”. **Values reported are based for male-associated factor positive patients only. Each variable considers a different denominator to calculate the percentages.

Husband Demographics			
Variables		All Samples [N (%) / Mean ± SD]	Male-associated factor positive^** ^[N(%) / Mean ± SD]
				Age (years)		35 ± 8	36 ± 9
Comorbidities^*^		44 (11%)	40 (19%)
Spermatic disease		39 (10%)	30 (14%)
Testicular disease		19 (5%)	13 (6%)
Diabetes		14 (4%)	12 (6%)
Hypertension		12 (3%)	10 (5%)
Others		44 (11%)	40 (19%)
Infertility problem*		136 (34%)	128 (62%)
Sperm motility (n = 275)		53 ± 32	36 ± 28
Normal morphology (n = 160)		6 ± 10	4 ± 9
Viscosity (n = 52)		0.3 ± 1	0.3 ± 1
Concentration (n = 313)		62 ± 108	22 ± 43
Volume (n = 310)		3 ± 3	2 ± 1
Abstinence days (n = 125)		4 ± 2	4 ± 1
Spermatocytes (n = 53)		2 ± 3	2 ± 4
Leukocytes (n = 51)		1 ± 2	1 ± 1
Received treatment^*^		55 (14%)	54 (26%)
Testicular sperm aspiration^*^		59 (15%)	57 (27%)
Testicular sperm extraction^*^		7 (2%)	7 (3%)

For the first cycle, the mean age and BMI were 30 ± 5 years and 29 ± 5, respectively. FSH supplementation was given to 320 women which can be sub-divided into: 72% (n = 231) human menopausal gonadotropin (HMG), 20% (n = 65) Gonal F, 7% (n = 23) Puragon. In addition, gonadotropin supplementation was given to 239 of the patients: 57% (n = 135) Decapeptil; 30% (n = 71) Cetrotide; 13% (n = 31) Lupron. The type of ART used was 54% (n = 208) ICSI, 37% (n = 45) IVF, and 9% (n = 35) split. The mean number of oocytes was (10 ± 6), cryopreserved (7 ± 2), and embryo transfer (3 ± 1). Please refer to Table 3 for further information regarding cycles one to five for all women.

**Table 3:** Clinical characteristics of all women based on each IVF cycle * Values based on categorical variable “Yes”.

Variable	Cycle 1 (n = 397)		Cycle 2 (n = 310)		Cycle 3 (n = 219)		Cycle 4 (n = 140)		Cycle 5 (n = 88)	
	N	Mean ± SD/%	N	Mean ± SD/%	N	Mean ± SD/%	N	Mean ± SD/%	N	Mean ± SD/%
Age	376	30 ± 5	127	31 ± 5	203	32 ± 5	132	33 ± 5	79	34 ± 4
BMI	339	29 ± 5	149	29 ± 5	170	29 ± 5	55	31 ± 5	35	31 ± 6
Number of Oocytes	371	10 ± 6	286	10 ± 6	195	11 ± 7	123	10 ± 6	81	9 ± 6
Number of Embryos Transferred	350	3 ± 1	269	3 ± 1	185	3 ± 1	114	3 ± 1	76	2 ± 1
Type of FSH	(320)		(260)		(171)		(105)		(73)	
Gonal F	65	20%	51	20%	36	21%	13	12%	13	18%
HMG	231	72%	184	71%	47	68%	82	78%	52	71%
Puragon	23	7%	24	9%	18	11%	9	9%	5	7%
Bravelle	1	0%	1	0%	1	1%	1	1%	3	4%
Type of GnRH	(239)		(203)		(135)		(87)		(58)	
Lupron	31	13%	34	16%	13	10%	11	13%	6	10%
Decapeptil	135	57%	114	56%	81	60%	49	56%	29	50%
Cetrotide	71	30%	52	26%	38	28%	26	30%	23	40%
Cycle Status*	(397)		(309)		(218)		(197)		(87)	
Done	358	90%	282	91%	191	88%	181	92%	82	94%
Cancelled	11	3%	6	2%	6	3%	7	4%	--	--
Failed	28	7%	21	7%	21	10%	9	5%	5	6%

**Figure 1:**
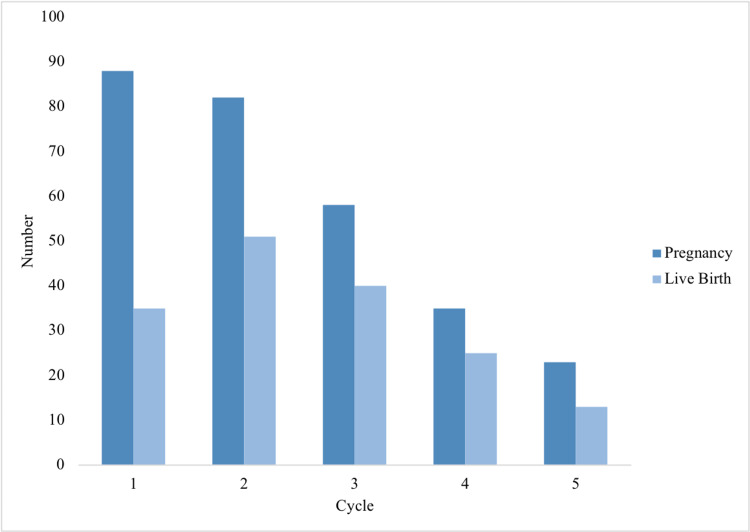
Comparison of total number of pregnancies vs. live birth across all cycles

**Figure 2:**
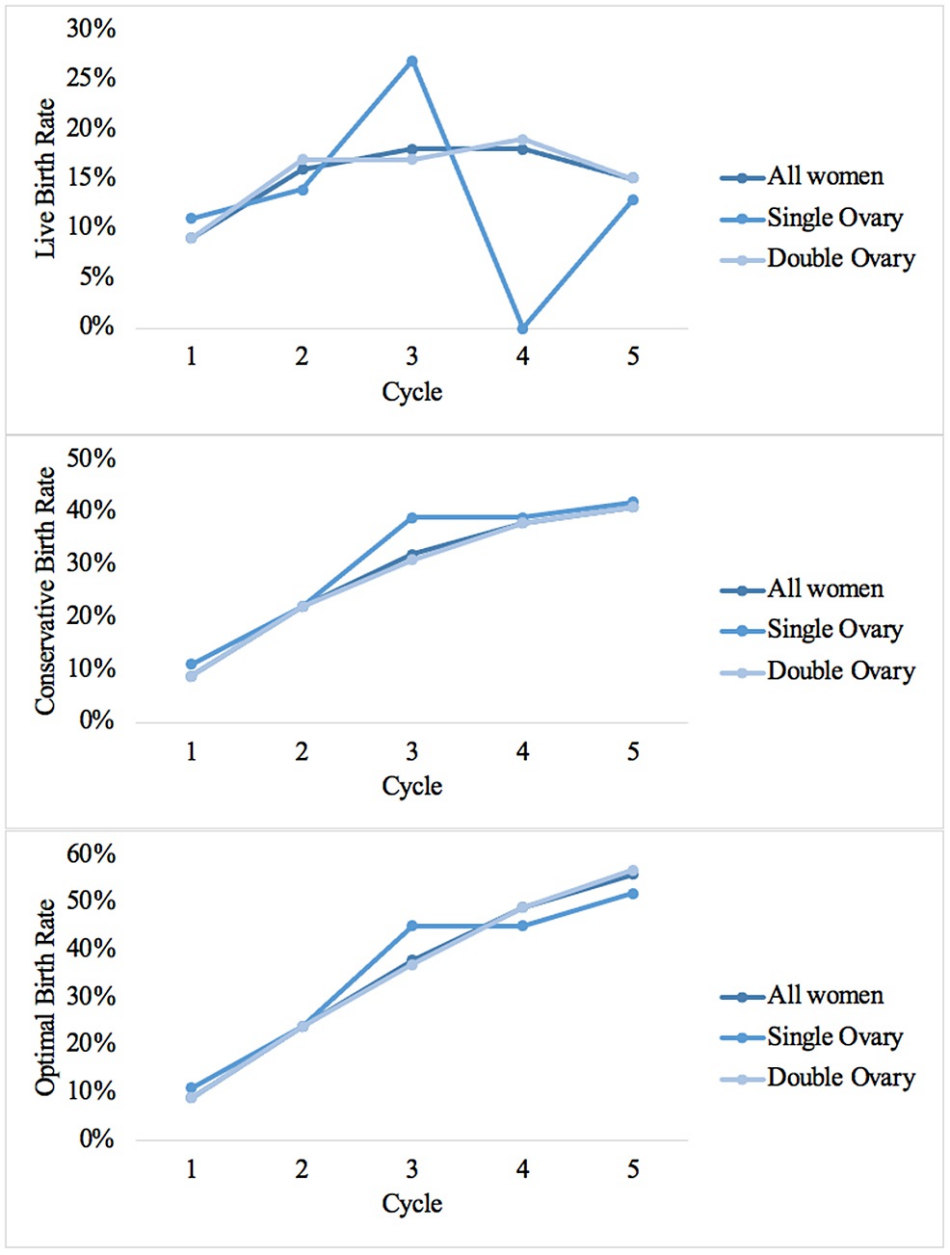
Live birth, conservative, and optimal birth rates in women with single vs. double ovary and all women

Live and cumulative birth rates (LBR and CLBR) were estimated for each cycle in Table 4. The total number of live births was 164. The live birth rates per cycle were 9%, 16%, 18%, 18%, and 15%, respectively. In the double ovary group, the highest rate was in the fourth cycle [19% (12-26)], while the single ovary group peaked in the third cycle [27% (9-46)]. We found no statistical difference between LBR and the number of ovaries (P > 0.05). Figure 1 shows a comparison between the number of pregnancies and live births in each cycle. Pregnancy was highest in the first cycle, accounting for 88 pregnancies, and lowest in the fifth cycle. The number of live births was highest in the second cycle and lowest in the fifth. In Figure 2, live, conservative, and optimal birth rates are presented in each cycle for all, double, and single ovary women. Live birth rates in both groups of women were similar in the first and second cycles and reached the highest rate for the single ovary group in the third cycle, while double ovary and all-women groups remained synchronous until the fifth cycle. In the fourth cycle, the single ovary group reached its lowest rate, which then increased in the fifth cycle.

**Table 4:** Live and cumulative birth rates across all cycles for 397 women undergoing IVF * Values are representing live birth rate in each cycle ** Conservative estimate of the cumulative live birth rate *** Optimal estimate of the cumulative live birth rate in three groups: all sample, single ovary, and double ovary.  Luke et al. and Major Greenwood formulas were used to estimate the rates, see appendix [14-15].

Cycle (i)	Number of women (N_i_)	Live births (LB_i_)	Live birth rate within each cycle, % (95% CI)*	Cumulative birth rates across all cycles % (95% CI)	
				Conservative estimate % (95% CI)**	Optimal estimate % (95% CI)***
1	397	35	9% (6-12)	9% (6-12)	9% (6-12)
2	310	51	16% (12-21)	22% (18-26)	24% (20-28)
3	219	40	18% (13-23)	32% (27-36)	38% (33-43)
4	140	25	18% (11-24)	38% (33-43)	49% (43-55)
5	88	13	15% (7-22)	41% (37-46)	56% (50-63)
Women with single ovary					
1	36	4	11% (1-21)	11% (1-21)	11% (1-21)
2	28	4	14% (1-27)	22% (9-36)	24% (9-38)
3	22	6	27% (9-46)	39% (23-55)	45% (23-62)
4	9	0	-----	39% (23-55)	45% (22-67)
5	8	1	13% (-10-35)	42% (26-58)	52% (28-75)
Women with double ovary					
1	361	31	9% (6-12)	9% (6-12)	9% (6-12)
2	282	47	17% (12-21)	22% (17-26)	24% (19-28)
3	197	34	17% (12-23)	31% (26-36)	37% (32-42)
4	131	25	19% (12-26)	38% (33-43)	49% (43-55)
5	80	12	15% (7-23)	41% (36-46)	57% (50-63)

## Discussion

Assisted reproductive technology (ART) has become the predominant treatment for infertility, leading to an increase in the number of infants born in most developed countries over the past 30 years. Many measures are used to express the effectiveness of ART, including LBR, defined as the live birth of a baby over 500 g or over 22 weeks of gestation if birth weight is unknown (deliveries of multiple pregnancies count as one live birth) [1]. Our study concentrates on the LBR after each cycle of treatment.

Both pregnancy rates and live birth rates are used to predict the success of ART, yet they differ based on many factors and do not directly correlate. Several studies explore these rates separately and identify possible factors such as age, embryo transfer duration, and number of oocytes [17]. In 2017, the Centers for Disease Control and Prevention (CDC), reported an LBR that ranged from 4.2% to 54.4% based on oocyte retrieval and an LBR that ranged from 11% to 48.5% based on embryo transfer [12]. Lind et al. found reduced LBR in women with a single ovary accounted for 18.6% compared with 25.4% in the double ovary group in the first cycle [10]. In our study, the LBR was higher in the single ovary group (11%) for the first cycle compared with the double ovary group (9%). A local study estimated the overall pregnancy rate as 35.1% (949 pregnancies) [18]. Here we found 286 pregnancies over 18 years and the highest number reached was 88 pregnancies in the first cycle.

In our comparison of women with one and two ovaries, the highest pregnancy rate occurred in different cycles in the two groups. However, the cycle-specific rates showed similar live birth rates among both single and double ovary women. Concordantly, a previous study also supports the argument that single ovary women have the same chances to conceive as a woman with two ovaries [19]. Similarly proven, another study underlined that the dose of HMG required, number of follicles seen upon ultrasound examination, number of obtained oocytes, number of embryos transferred, and pregnancy rates were not statistically different between both groups of women. Nevertheless, double ovary women tend to have more follicles aspirated during laparoscopy [20].

Our results demonstrated that the average age of women seeking ART was 32 ± 5 years, which is in accordance with findings reported by studies performed locally and internationally [18,21]. They were divided into primary infertility (59%) and secondary infertility (41%), which are also consistent with two other studies that show a higher prevalence of primary infertility [18,22]. The highest incidence of female comorbidities was hypothyroidism (17%), followed by hyperprolactinemia (8%). According to the literature, an early miscarriage can be caused by a decrease in thyroxin levels, and a retrospective cohort study showed non-significant differences in the CLBR between women treated for hyperprolactinemia and control groups [21,23]. These two findings highlight the importance of pre-cycle treatment. On the other hand, the most common male comorbidities were spermatic and testicular-related disease (19% and 14%). A single-center control study compared the prevalence of comorbidities between infertile and fertile men, revealing a significantly higher percentage in the infertile men, particularly hypertension (17.8% vs. 7.1%) [24]. A similar pattern was observed in this study (6% vs. 4%).

Female-associated factors accounted for 48% of the infertility causes; the most common cause was motility (44%), followed by tubular factors (27%), which was previously reported to be the second most common factor in female infertility [25]. Male-associated factor infertility was the most common indication for ART, accounting for 52% of the causes. This is consistent with what has been found in previous studies [18,26]. Semen quality is a measure of male infertility; therefore, up to 90% of infertility cases are due to sperm abnormalities, which could stem from testicular factors [27-28]. Cases with male-associated infertility factors were grouped into two categories: spermatic diseases (19%) and testicular diseases (14%). Women with male-associated infertility factors had the highest pregnancy rate using in vitro fertilization [29].

Limitations

This is a single center study and the generalizability of the results are restricted. However, the research provides insight into the less studied comparison of birth rates among single and double ovary women. King Abdulaziz Medical City is one of the busiest IVF centers in Saudi Arabia and receives patients from all over the country, and many patients have been lost in the final analysis due to loss of follow-up. The results of this study hold importance in the context of Saudi population. All of the estimates in this study were reported based on the number of ovaries. The different causes for infertility were not considered in the calculation of estimates as it was outside of the scope of the present study. Also, all of the patients were undergoing ART, therefore, the probability of getting pregnant irrespective of the cause of infertility was assumed to be the same for all patients. Future studies that include multiple centers in more than one region of Saudi Arabia are recommended, which will be a better reflection on current practice.

## Conclusions

In conclusion, the outcomes of ART varied between studied groups and different factors contributed to the outcomes. Live birth rates were lower in single ovary women. The average across five cycles in the single and double ovary groups were estimated as 13% and 15%, respectively, with higher numbers of live births recorded among those with two ovaries. There was no statistical difference in live birth rate between single and double ovary women. The difference between pregnancy and live birth numbers was drastic in the first cycle. We recommend optimizing the quality of care by increasing patient education as well as thorough observation, and regular follow-ups through the cycle and pregnancy period to prevent unfortunate events. Further studies to explore the drop in live birth rate among single ovary women are highly recommended.

## Appendices

Supplementary file

The linking of women to each cycle was done based on the information available for each patient in their medical record files. Irrespective of the number of cycles the women had, each cycle information was extracted after checking the MRN number and counterchecking it with the date of birth and husband's name to make sure the cycles were linked with the corresponding patients. In cases where there were discrepancies, all the files were physically checked as hard copies

The estimation of the birth rate was done using the following formulas:

A.   Conditional Live Birth Rate (pi) = Number of live births / Number of women

                                               = LBi / Ni

                                    SE(pi)  = [pi * (1-pi) / Ni]½  

Where LBi refers to births ending in viable babies (>22 weeks of gestation), Ni is number of all eligible women for the corresponding cycle, and SE is the standard error.

B.    Conservative cumulative live birth rate (CCLi) = Cumulative live births / Number of women at 1st cycle 

                                                                        = [ ∑j=1,i LBj ] / Ni

                                                             SE(CCLi) = [CCLi *(1 - CCLi) / N1]½

Where Ni is the number of women who had live birth from the 1st cycle to cycle i, LB is the live births, and SE is the standard error.

C.    Optimal cumulative livebirth rate (OCLi) = OCLi-1 + pi *(1 - OCLi-1) 

                                                                   (Product limit estimate)

                                                   SE(OCLi) = (1 - OCLi) * [∑j=1,i pj / (Nj - LBj)]½ 

                                                                   (Greenwood’s formula)

Where OCLi was based on the product limit estimate also known as the Kaplan-Meier estimator. Greenwoods’s formula was used to put the SE (standard error) on the Kaplan Meier estimator [14-15].

## References

[1] Zegers-Hochschild F, Adamson GD, Dyer S (2017). The international glossary on infertility and fertility care, 2017. Fertil Steril.

[2] Vander Borght M, Wyns C (2018). Fertility and infertility: definition and epidemiology. Clin Biochem.

[3] (2018). The World Bank. Fertility rate, total (births per woman). https://data.worldbank.org/indicator/SP.DYN.TFRT.IN.

[4] Ishikawa M (1995). Assisted reproductive technology; IVF-ET [Article in Japanese]. Hokkaido Igaku Zasshi.

[5] Bhandari HM, Choudhary MK, Stewart JA (2018). Complications of assisted reproductive technology treatment and the factors influencing reproductive outcome. Obstet Gynaecol.

[6] de Carvalho BR, Rosa e Silva AC, Rosa e Silva JC, dos Reis RM, Ferriani RA, Silva de Sá MF (2008). Ovarian reserve evaluation: state of the art. J Assist Reprod Genet.

[7] Rouzi AA, Sahly N, Kafy S, Sawan D, Abduljabbar H (2015). Prevalence of endometriosis at a university hospital in Jeddah, Saudi Arabia. Clin Exp Obstet Gynecol.

[8] Khan Z, Gada RP, Tabbaa ZM, Laughlin-Tommaso SK, Jensen JR, Coddington CC 3rd, Stewart EA (2014). Unilateral oophorectomy results in compensatory follicular recruitment in the remaining ovary at time of ovarian stimulation for in vitro fertilization. Fertil Steril.

[9] Taheripanah R, Zamaniyan M, Meybodi MK, Amir-Arjmand MH, Mansouri A, Taheripanah A, Malih N (2019). Are intra follicular estradiol and oocytes quality in women undergoing assisted reproductive technology different between the right and left ovaries? An observational study. Eur J Obstet Gynecol Reprod Biol X.

[10] Lind T, Holte J, Olofsson JI (2018). Reduced live-birth rates after IVF/ICSI in women with previous unilateral oophorectomy: results of a multicentre cohort study. Hum Reprod.

[11] De Neubourg D, Bogaerts K, Blockeel C (2016). How do cumulative live birth rates and cumulative multiple live birth rates over complete courses of assisted reproductive technology treatment per woman compare among registries?. Hum Reprod.

[12] (2021). Department of Health U, Services Centers for Disease Control H. 2017 Assisted reproductive technology fertility clinic success rates report. Control H. 2017 Assisted Reproductive Technology Fertility Clinic Success Rates Report [Internet.

[13] Abuzeid MI, Bolonduro O, La Chance J (2014). Cumulative live birth rate and assisted reproduction: impact of female age and transfer day. Facts Views Vis Obgyn.

[14] Luke B, Brown MB, Wantman E (2012). Cumulative birth rates with linked assisted reproductive technology cycles. N Engl J Med.

[15] Greenwood M Jr (1926). The natural duration of cancer. Reports of Public Health and Related Subjects.

[16] Smith AD, Tilling K, Nelson SM, Lawlor DA (2015). Live-birth rate associated with repeat in vitro fertilization treatment cycles. JAMA.

[17] Cetin MT, Kumtepe Y, Kiran H, Seydaoglu G (2010). Factors affecting pregnancy in IVF: age and duration of embryo transfer. Reprod Biomed Online.

[18] Almaslami F, Aljunid SM, Ghailan K (2018). Demographic determinants and outcome of in vitro fertilization (IVF) services in Saudi Arabia. J Int Med Res.

[19] Lass A (1999). The fertility potential of women with a single ovary. Hum Reprod Update.

[20] Alper MM, Seibel MM, Oskowitz SP, Smith BD, Ransil BJ, Taymor ML (1985). Comparison of follicular response in patients with one or two ovaries in a program of in vitro fertilization. Fertil Steril.

[21] Duan Y, Liu X, Hou W (2019). No impact of treated hyperprolactinemia on cumulative live birth rate and perinatal outcomes in in vitro fertilization-embryo transfer. J Obstet Gynaecol Res.

[22] Al-Turki HA (2015). Prevalence of primary and secondary infertility from tertiary center in eastern Saudi Arabia. Middle East Fertil Soc J.

[23] Kawwass JF, Crawford S, Session DR, Kissin DM, Jamieson DJ (2015). Endometriosis and assisted reproductive technology: United States trends and outcomes 2000-2011. Fertil Steril.

[24] Shiraishi K, Matsuyama H (2018). Effects of medical comorbidity on male infertility and comorbidity treatment on spermatogenesis. Fertil Steril.

[25] Kumar P, Mohan S, Talwar P, Rai S, Nagaraja N, Sharma P (2017). Diagnostic office vaginohysteroscopy in evaluation of infertility prior to IVF: a retrospective analysis of 1000 cases. J Obstet Gynaecol India.

[26] Stern JE, Luke B, Tobias M, Gopal D, Hornstein MD, Diop H (2015). Adverse pregnancy and birth outcomes associated with underlying diagnosis with and without assisted reproductive technology treatment. Fertil Steril.

[27] Glazer CH, Bonde JP, Giwercman A, Vassard D, Pinborg A, Schmidt L, Vaclavik Bräuner E (2017). Risk of diabetes according to male factor infertility: a register-based cohort study. Hum Reprod.

[28] Sabra SM, Al-Harbi MS (2014). An influential relationship of seminal fluid microbial infections and infertility, Taif Region, KSA. World J Med Sci.

[29] Mukhtar HB, Shaman A, Mirghani HO, Almasalmah AA (2017). The outcome of assisted reproductive techniques among couples with male factors at Prince Khalid Bin Sultan fertility centre, Kingdom of Saudi Arabia. Open Access Maced J Med Sci.

